# A protocol for the development of a core descriptor and core outcome set for acute left‐sided diverticulitis

**DOI:** 10.1111/codi.70645

**Published:** 2026-10-10

**Authors:** CODD‐ Collaborative for Outcomes in Diverticular Disease, S Lawday, A Fahey, Z Khan, D Vimalachan, MJ Lee, A Hawkins, M Lee

**Keywords:** descriptors, diverticulitis, outcomes, protocol

## Abstract

**Background:**

Diverticulitis is a common emergency general surgical condition that affects patients worldwide. Research remains heterogeneous, meaning the provision of high‐quality, evidence‐based care for these patients remains a challenge. We aimed therefore to complete a core outcome and core descriptor set for acute left‐sided diverticulitis.

**Methods:**

An international team set out to develop a COS and CDS using Core Outcome Measures in Effectiveness Trials (COMET) guidance.

The production of a longlist of domains will initially be completed. This will initially involve a systematic review to identify different descriptors and outcomes. Patient engagement through a minimum of two focus groups with patients will be completed to ensure the completeness of the final lists. The final lists of descriptors and outcomes will then be prioritised utilising a sequential, multi‐round international electronic Delphi of healthcare professionals, with patients contributing to the COS development. A final consensus meeting will then be held to ratify the final components of the COS.

**Dissemination:**

The results of this COS and CDS will be presented internationally at conferences and published in open access journals. Further dissemination through professional networks, the executive group and patient groups utilising patient friendly material will also be completed.

**Significance statement**

Diverticulitis is a common emergency surgical presentation affecting up to 4% of the population. The need for core outcome sets to standardise and improve research is well established. This project aimed to complete a high‐quality diverticulitis core outcome set and core descriptor set.

## INTRODUCTION

Diverticulitis is a common general surgical condition characterised by inflammation of diverticula, or outpouchings of the colon, which affects patients worldwide. While diverticula and subsequent diverticulitis can occur anywhere in the colon, it most commonly affects the left colon in Western countries [1]. Currently, research into left‐sided diverticulitis, its treatments and outcomes is heterogeneous, meaning the provision of high‐quality, evidence‐based care for these patients remains a challenge.

The variability in current research, while fostering innovation and diverse intervention strategies, hampers generalisability across studies, makes communication and reporting amongst researchers difficult and restricts the ability for high‐quality meta‐synthesis [2]. Therefore, it has become increasingly important to develop a core set of outcomes and descriptors that integrate current literature. This process should be guided by COMET (Core Outcome Measures in Effectiveness Trials), a set of guidelines designed for the development, implementation, evaluation and updating of core outcome sets (COS) [3].

Currently, there exists no published COS or core descriptor set (CDS) for left‐sided diverticulitis in the literature. Given the heterogeneity in the research and reporting of outcomes and descriptors for diverticulitis and its patients, we aimed to develop a CDS and COS for acute left‐sided diverticulitis to standardise reporting and communication on this disease process for future studies.

## SCOPE

The CDS and COS will be utilised for patients with acute left‐sided diverticulitis regardless of treatment strategy. Although there will be outcomes and domains relevant to patients with right‐sided colonic diverticulitis, this is not the focus of this work. This should be relevant to all interventions (medical and surgical or radiological). The context for use will be research on patients with acute left‐sided diverticulitis in the acute hospital/secondary care setting.

## AIM

The aim of this project was to co‐develop a comprehensive core outcome and CDS for patients suffering from acute left‐sided diverticulitis.

## OBJECTIVES

- Generate and extract a longlist of domains through systematic searches of the published literature.
- Utilise patient focus groups to ratify and assess completion of the domain longlist.
- Gain international consensus on which domains should be included in both the core descriptor and core information sets via Delphi methodology [4].
- Finalised the acute left‐sided diverticulitis CDS and COS in a final consensus meeting.

## METHODS

The same methodology, as described below, will be utilised to create the CDS and the COS. We will adhere to methods for COS development outlined in the COMET handbook and Core Outcome Set‐STAndards for Development guidelines. This protocol has been developed utilising the COS‐STAP statement [5]. The definitions used for acute diverticulitis were taken from international consensus work previously completed by the same collaborator group.

The executive group for the CDS and COS development has been formed by the subspeciality lead (SSL) and associate subsueciality lead (aSSL) for emergency general surgery as appointed by the Association of Surgeons of Great Britain and Ireland and the Royal College of Surgeons (England), alongside the chief investigator of an international cohort study on diverticulitis recently published in the Lancet and an Associate Professor from the United States with post‐graduate qualifications from Harvard and diverticulitis researchers and their respective research associates.

### Stakeholders

Stakeholders have been identified as part of the work‐up process for this CDS and COS. Surgeons and gastroenterologists have been actively engaged to ensure international coverage of those involved. Surgeons have been engaged through the Association of Surgeons of Great Britain and Ireland, ASCRS (American Society of Colon and Rectal Surgeons), Association of Coloproctology of Great Britain and Ireland, European Society of Coloproctology and social media channels. Gastroenterologists have been engaged through the British Society of Gastroenterology. Pre‐existing patient groups for diverticulitis exist through the national charities Bowel Research UK and Guts UK.

The methodology for the CDS and COS is set out in Figure 1.

**FIGURE 1:**
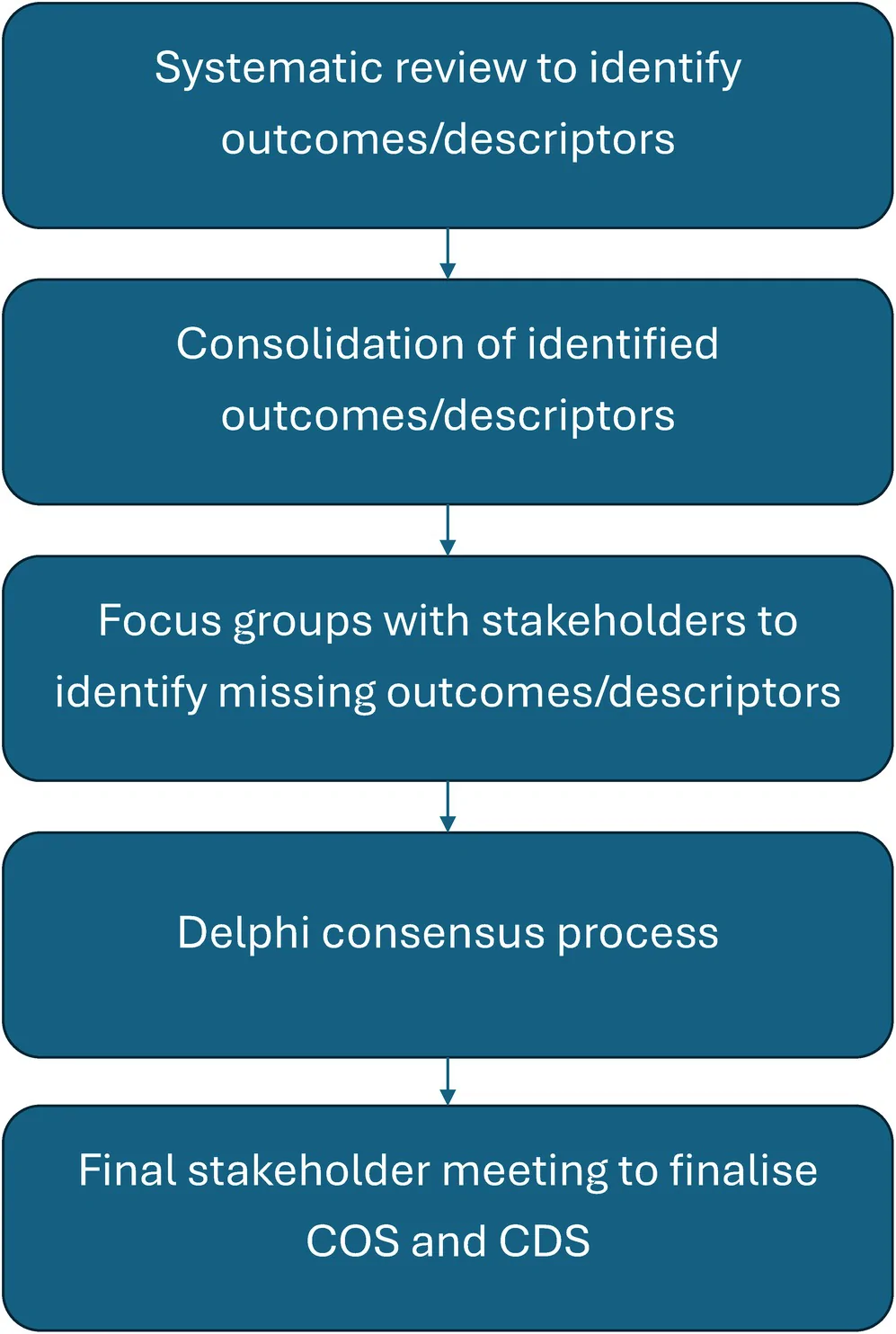
Flowchart of core outcome and descriptor set methodology.

### Information sources

A systematic review will be completed to identify relevant literature on acute left‐sided diverticulitis. The search strategy can be seen below in Table 1. Papers will only be included if they have been published in the last 5 years. Papers will only be included if they are available in the English language.

**TABLE 1:** Search strategy.

1	Diverticulitis, Colonic/
2	(Sigmoid Diseases/or Colon, Sigmoid/) and Diverticulitis/
3	Diverticulitis/
4	(Diverticulitis* or diverticulosis*).ti.
5	((sigmoid* or acute or uncomplicat* or un‐complicate*) adj4 diverticul*).ti.
6	1 or 2 or 3 or 4 or 5
7	*Anti‐Bacterial Agents/
8	(antibiotic* or anti‐bacterial* or antibacterial* or anti‐mycobacterial* or antimycobacterial* or bacteriocidal* or bacteriocide*).ti,ab.
9	(non‐antibiotic* or nonantibiotic*).ti,ab.
10	Drainage/
11	(drain or drainage or drains or drained).ti,ab.
12	(surgery or surgical).ti,ab.
13	or/7–12
14	(outcome* or safety or efficacy or management*).ti,ab.
15	6 and 13 and 14
16	limit 15 to (english language and yr. = “2010 ‐Current”)
17	editorial/or news/or exp. historical article/or anecdotes as topic/ or case reports/
18	(letter or comment*).ti.
19	(abstract or comment or conference or letter or chapter).pt.
20	17 or 18 or 19
21	16 not 20

Inclusion criteria:

- Undergoing treatment for acute left‐sided diverticulitis.
- Study designs:
  ○ Retrospective cohort.
  ○ Prospective cohort.
  ○ Randomised controlled trial.
- Reporting on clinical outcomes of diverticulitis/diverticular.

Exclusion criteria:

- Case reports.
- Translational studies.
- Animal studies.
- Studies not focused on diverticular disease.
- Systematic reviews and narrative reviews.
- Clinical practice guidelines.
- Diverticular bleeding only.
- Right‐sided diverticular disease only.

Abstracts will undergo dual screening using the Covidence software (SL, AF, ZK, DV, ML and AH). Dual‐author full‐text screening will be completed to identify papers published on patients who are diagnosed with acute left‐sided diverticulitis (SL, AF and AH).

The expectation is that saturation will be reached before all paper identified within the review undergo data extraction. Therefore, papers will then be listed in a chronological order, from most recent. The first 40 papers will undergo full data extraction for outcomes and descriptors by two authors, independently. Outcomes and descriptors will be extracted into an online file. Following this, domains will be created by grouping similar descriptors and outcomes, with this completed by a single author but checked by the wider executive group to ensure consensus is made, with disagreement resolved through discussion. This will be completed by combining similar outcomes or descriptors into a single heading, for example abscess formation and Hinchey grade.

Following the completion of this process, a further four papers (10% of included papers) will undergo extraction to see whether any further descriptors or outcomes are identified. This will be repeated until no substantial new domains are identified. The extracted outcomes and domains will then be consolidated within the executive group to combine similar outcomes (i.e., time spent in hospital, length of hospital stay or sepsis, septic shock, and septicaemia). The outcomes and descriptors will then be grouped together by the executive group. The final groups will be dependent on the outcomes and descriptors identified. However, it is anticipated that the descriptors will be grouped into, but not limited to, patient demographics, diverticular disease details and investigations. Outcomes will also be grouped into, but again not limited by, surgical outcomes, medical outcomes, recovery outcomes and follow‐up outcomes. The grouping of both the outcomes and descriptors will be an iterative process within the executive group.

This list of domains will then be discussed at a minimum of two international patient focus groups. Patient representatives will be given the lists of domains in advance and will be invited to contribute to and assess the completeness of the lists. These focus groups will aim to be facilitated by at least one member of the executive group. Patients in the UK will be identified through pre‐existing patient groups via GUTS UK and patients in the United States will be invited from a pre‐existing patient advisory board.

### Consensus process

An international online multi‐round Delphi survey will then be completed. Domains generated as part of the literature work and focus groups will all be listed. This will be completed using an appropriate online software (Research electronic data capture [REDCap], a secure, web‐based software platform). Draft surveys will be piloted within the executive group and shown to patient groups to ensure understandability. This survey will be in English, with multi‐lingual surveys being outside of the scope of the resources of this project and concerns remaining amongst the executive group that translation may lead to subtle changes of language which could lead to conceptual inconsistency.

A minimum of two Delphi rounds will be undertaken, with participants separated by background (researcher, clinician—surgeon, clinician—gastroenterologist, patient). This will be done to understand any differences in priorities and avoid over‐representation. Some background demographic details of the participants will be collected during the initial Delphi round. The COS will be circulated amongst all stakeholders, with the CDS only circulated amongst professional stakeholders.

We will recruit using a purposive sample of subject‐matter experts using an established professional network of clinicians, researchers and stakeholders with relevant expertise. This network‐based recruitment will be supplemented through outreach via national professional society connections and targeted announcements on professional social media platforms (e.g. X/LinkedIn). Patients will be recruited through pre‐existing patient groups, PPIU groups of professional organisations and social media.

During each round, participants will be asked to rate the importance of each domain against a 9‐point Likert scale based upon the Grading of Recommendations, Assessment, Development and Evaluations (GRADE) scale. This will be labelled as not essential at 1 to essential at 9. A free box will allow for comments and new missing domains to be suggested. New domains will be included in subsequent rounds of the Delphi if they are suggested by more than 10% of participants or at the request of the executive group. Only those who have completed Round 1 will then be invited to Round 2, and this will continue with any further rounds.

### Consensus definitions

Consensus definitions for the first round are described below. These may be slightly modified for subsequent Delhi rounds if required, and this will be done by consensus within the executive group. These definitions have been utilised in other outcome sets completed by members of the executive committee [6].

- Consensus IN is defined as >70% of participants rating the outcome domain as 7–9 and < 15% rating the outcome domain as 1–3. This means the outcome domain will be taken forward to the consensus process. If more than 90% of a single group rate this as 7–9, this will be taken forward.
- Consensus OUT is defined as >70% of participants rating the outcome domain as 1–3 and < 15% rating the outcome domain as 7–9. This means the outcome domain will not be taken forward to the consensus meeting. If more than 90% of a single group rate this as 1–3, this will not be taken forward.
- All other combinations of response results are defined as ‘NO CONSENSUS’.

### Analysis

#### Outcome

International consensus meeting will be held following the completion of the Delphi surveys. The number of meetings will be determined by the executive committee following completion of the surveys with attempts that timing and geography of these meetings allow for diverse international stakeholder engagement. These meetings will utilise nominal group technique.

This meeting will involve structured discussion about each proposed included domain and any excluded domain that require further discussion (e.g. meeting consensus OUT but being identified as important by a particular subgroup [patients]). Interactive, anonymous voting will take place to determine final inclusion and exclusion of domains in the final CDS and COS.

#### Missing data

All questions will be mandatory to reduce missing data, although there will be an unsure button if a participant does not know how to grade a specific domain.

## AUTHOR CONTRIBUTIONS

**CODD‐ Collaborative for Outcomes in Diverticular Disease:** Conceptualization; investigation; writing – original draft; methodology; writing – review and editing; supervision.

## FUNDING INFORMATION

Samuel Lawday is an NIHR Doctoral Research Fellow (NIHR303276). The views expressed are those of the author(s) and not necessarily those of the NIHR or the Department of Health and Social Care.

## CONFLICT OF INTEREST STATEMENT

The authors declare no conflicts of interest.

## DISCLOSURE

The final CDS and COS will be presented at various international conferences, with submission planned to ACPGBI, ASCRS, ESCP and ASGBI. Publication is planned in an open access publication. Further dissemination through social media, professional organisations and patient groups is planned.

S Lawday (Centre for Surgical Research, Bristol Medical School, University of Bristol, Bristol, UK), A Fahey (Vanderbilt University School of Medicine, Nashville, TN, USA, Section of Colon & Rectal Surgery and Vanderbilt University Medical Center, Nashville, TN, USA), Z Khan (Royal Liverpool Univeristy Hospital, Liverpool University Hospitals NHS Foundation Trust), D Vimalachan (Institute of Systems, Molecular and Integrative Biology, University of Liverpool, Liverpool, England), MJ Lee (University of Birmingham & University Hospitals Birmingham, Birmingham, England), A Hawkins (Vanderbilt University School of Medicine, Nashville, TN, USA and Section of Colon & Rectal Surgery, Vanderbilt University Medical Center, Nashville, TN, USA).

## ETHICS STATEMENT

All research related activities will be conducted under the supervision of appropriate institutional review boards.

## ETHICS APPROVAL

Ethics approval for PPI focus groups was not required in the UK; however, IRB approval was completed in the United States.

## Data Availability

Data sharing not applicable to this article as no datasets were generated or analysed during the current study.
